# Malformation of the Thorax

**Published:** 1835

**Authors:** 

**Affiliations:** Kentucky


III.—*Malformation of the Thorax.*—Dr. INGELS, of Kentucky, has given, in the Transylvania Journal, a statement of the following singular malformation of the thorax, in a negro child, which died at the age of eighteen months; during which its general health was good, although the abnormal structure was constantly increasing. It appeared to die of suffocation, gradually induced.

"The peculiarity of the case consisted mainly in an apparent effort, on the part of nature, to separate the cavity of the chest into two apartments—an anterior and a posterior. This was measurably accomplished by a very extensive incurvation of the nine or ten superior ribs, on each side; so that, at the distance of about one third of their length from the sternal ends, (the point of greatest indentation,) their internal surfaces were very considerably approximated. But the division was rendered much more complete by large cartilaginous tubercles, of a semi-spherical form; one of which was based upon the point of the internal convexity of each curved rib. These tubercles were ranged in vertical rows, one on each side, and equidistant from the sternum. They presented a singular and beautiful appearance; and when discovered, the case assumed a new and more interesting character.

"The heart presented itself immediately to view, when the sternum was elevated. It occupied the anterior chamber, to the exclusion of the lungs; these being thrown into the posterior, were not brought into view, until the heart was partially removed, and the approximated ribs forced wider apart. As the heart was relatively large, and the pericardium contained about three ounces of a serous fluid, they quite filled up the front cavity. The cavities of the heart, in this instance, might have been very appropriately designated by the terms, right and left; for the apex of the organ pointed to the sternum, while its base looked to the vertebral column. Both the lungs and heart were in a healthy condition; the right cavities of the latter were more than usually distended, and the lungs were very much engorged with blood.—There was, however, the utmost disparity in volume, between the heart and lungs—the latter being exceedingly defective in size. The abdominal viscera were examined; they appeared to be healthy; the spleen only seeming to be preternaturally firm, and like the liver in appearance. It was, however, very obvious, that the abdominal viscera, generally, were out of all proportion to those of the chest. There was as much relative disparity between them, as was observed between the heart and lungs. The liver was astonishingly large; the mesenteric glands in particular were very numerous, and of great comparative magnitude. The stomach was unusually capacious, and I have, since the examination, thought that the small bowels were uncommonly voluminous."—P. 488—9.

